# Occurrence of *Trichinella* spp. in Grey Wolves and Red Foxes: Insights from Wild Mammal Surveillance in Emilia-Romagna (Italy)

**DOI:** 10.3390/ani15243532

**Published:** 2025-12-08

**Authors:** Camilla Torreggiani, Chiara Garbarino, Giovanni Pupillo, Giorgia De Lorenzi, Maria Sampieri, Elisa Massella, Gianluca Rugna, Alessandro Reggiani, Silva Rubini, Matteo Frasnelli, Letizia Cirasella, Giorgio Galletti, Gianluca Marucci, Francesco Celani, Giulia Maioli

**Affiliations:** 1 Istituto Zooprofilattico Sperimentale Della Lombardia e Dell’emilia Romagna “Bruno Ubertini”, Via Bianchi 9, 25124 Brescia, Italy; 2 Unit of Foodborne and Neglected Parasitic Diseases, Department of Infectious Diseases, Istituto Superiore di Sanità, Viale Regina Elena 299, 00161 Rome, Italy

**Keywords:** *Trichinella britovi*, wild mammals, surveillance

## Abstract

*Trichinella* spp. are zoonotic nematodes with a global distribution, primarily maintained through wildlife reservoirs which complicates eradication efforts. In Europe, four species—*T. spiralis*, *T. britovi*, *T. nativa*, and *T. pseudospiralis*—circulate in wild carnivores and omnivores, with *T. britovi* and *T. spiralis* being the most prevalent in wild and domestic hosts. This study aimed to assess the presence and species distribution of *Trichinella* spp. in wild mammals. This study presents the results of five years of wildlife surveillance carried out within the long-standing monitoring program implemented in the Emilia-Romagna region, northern Italy, which has been in place since 2006. Between 2020 and 2024, a total of 104,338 wild mammals, including red foxes, wolves, and wild boar, were tested using the magnetic stirrer digestion method in accordance with EU regulations. A total of 12 animals (0.011%) were found to be infected with *Trichinella* larvae. Molecular identification of positive samples, performed at the European Union Reference Laboratory for Parasites, showed the presence of *T. britovi* in 11 animals, while one isolate remained unidentified. *T. britovi* was detected primarily in wolves and red foxes. No infections were identified in wild boar during the study period. Overall, the low prevalence observed confirms that *Trichinella* spp. infections in wildlife in Emilia-Romagna are sporadic but persistently present. Only *T. britovi* was detected, supporting its role as the predominant species in this area. These findings highlight the importance of long-term surveillance for early detection and risk assessment within a One Health framework.

## Simple Summary

*Trichinella* spp. are zoonotic parasites transmitted through the consumption of raw or undercooked meat containing infective larvae. These nematodes circulate mainly among wild carnivores and omnivores, occasionally infecting domestic animals and humans. Although Italy is considered a low-risk country, sporadic outbreaks linked to game meat still occur. This study reports five years (2020–2024) of wildlife surveillance in the Emilia-Romagna region, northern Italy, aimed at assessing the presence of *Trichinella* spp. in wild mammals. A total of 104,338 animals—including wild boars, red foxes, and wolves—were examined using the magnetic stirrer digestion method. *Trichinella* larvae were detected in 12 animals (0.011%); seven wolves and four red foxes. Molecular identification revealed *Trichinella britovi* in 11 of them, confirming it as the only species currently circulating in the area. No infections were detected in wild boars. These findings indicate that *Trichinella* infections in wildlife in Emilia-Romagna are rare but persistently present. Wolves and red foxes represent the main reservoirs maintaining the sylvatic cycle of *T. britovi*. The continued absence of *Trichinella* in wild boar suggests a limited role for this species locally. Long-term surveillance remains essential for early detection, food safety, and zoonotic risk assessment within a One Health framework.

## 1. Introduction

*Trichinella* spp. are parasitic nematodes with a global distribution and the ability to infect a wide range of host species. Despite typically having a low prevalence in wildlife, these hosts are essential for sustaining the parasite’s natural transmission cycle within ecosystems [1]. Wildlife reservoirs may facilitate the spillover of *Trichinella* spp. to domestic animals, particularly pigs, and occasionally to other species such as horses, thereby posing a potential risk to human health. As a strictly foodborne pathogen, transmission occurs exclusively through the ingestion of raw or undercooked meat containing viable larvae. Consequently, carnivorous, omnivorous, and scavenging species are the most commonly affected [1]. Moreover, anthropogenic activities—including the improper disposal of infected carcasses or offal from domestic pigs and hunted wildlife—can significantly enhance transmission opportunities at the wildlife–livestock–human interface. Within Europe, four main species of *Trichinella* are reported among wild carnivores: *T. spiralis*, *T. britovi*, *T. nativa*, and *T. pseudospiralis* [2]. Several of these species are capable of infecting both wild and domestic pigs. Apex predators and scavengers, notably the red fox (*Vulpes vulpes*) and the wolf (*Canis lupus*), are key players in maintaining the sylvatic cycle of *Trichinella*. Among these hosts, along with wild boar (*Sus scrofa*), *T. britovi* and *T. nativa* are the most frequently identified species, while *T. spiralis* and *T. pseudospiralis* occur less commonly [2,3]. *T. britovi* exhibits a wide distribution across wild carnivore populations throughout continental Europe, western Asia, and parts of northwestern Africa, though it is generally absent from insular environments. *T. spiralis*, the first species described within the genus, has received considerable attention due to its major role in trichinellosis and its utility as a model organism in parasitology. It is known for its high infectivity and the long-term viability of larvae within the muscle tissues of both wild and domestic pigs, where they can survive for over three years [4]. Consequently, *T. spiralis* remains the principal etiological agent of trichinellosis worldwide [5], although it continues to circulate in wildlife populations that serve as a secondary reservoir.

Italy is considered a country with low incidence of trichinellosis, largely due to strict meat inspection programs and veterinary surveillance; however, sporadic outbreaks continue to occur, mainly linked to the consumption of raw or undercooked meat products derived from wild boar or free-ranging pigs. The most recent outbreak was reported in northwestern Italy in 2019–2020, when 35 individuals developed trichinellosis after consuming raw sausages prepared from the meat of a single wild boar, with *T. britovi* identified as the etiological agent. This event highlighted the epidemiological role of wild boar as the main wildlife reservoir of *Trichinella* spp. in Italy, particularly in terms of transmission to humans and domestic cycles, and emphasised the public health risks associated with traditional practices of consuming untreated game meat [6]. Outbreaks associated with domestic pigs occurred in Sardinia in 2005, 2006, and 2011, involving a total of 31 individuals who consumed raw sausages made from free-ranging sows. In all cases, the causative agent was *T. britovi.* This episode was of particular epidemiological significance, as it challenged the assumption that some Italian regions, such as Sardinia, were “*Trichinella*-free,” and underscored the risks associated with non-intensive farming systems and home-slaughtering practices [7]. Both outbreaks confirm that raw pork products remain the primary vehicle for human infection in Italy and that *T. britovi* is the most common species involved in the Mediterranean area, reflecting its wide distribution among wildlife reservoirs such as foxes, wolves, and wild boars. These findings underscore the importance of maintaining high levels of epidemiological vigilance, promoting consumer education on the safe consumption of meat, and ensuring rigorous veterinary controls even in areas traditionally considered low-risk.

In light of the importance of wildlife in the epidemiology of *Trichinella* spp., this study presents the outcomes of an extensive monitoring program conducted from 2020 to 2024, focusing on wild mammalian hosts within the Emilia-Romagna region of northern Italy. It presents a current overview of the results from the ongoing wildlife monitoring program established in 2006.

## 2. Materials and Methods

### 2.1. Study Area

The study was conducted in Emilia-Romagna, a region in northern Italy covering approximately 22,500 km^2^ and extending from the Po river plain to the northern Apennines. The area includes a mosaic of intensive agricultural landscapes, peri-urban zones, forested hills, and protected natural parks. This environmental heterogeneity supports a diverse wildlife community and favours frequent interactions between wild species, livestock, and humans, thereby creating relevant conditions for pathogen circulation and cross-species transmission.

Since 2006, Emilia-Romagna has implemented a regional wildlife health surveillance program aimed at the early detection of priority pathogens—particularly zoonotic agents—and at improving knowledge on wildlife health status to support risk assessment for livestock and public health. The program was initially established in response to the requirements of EU Regulation 2075/2005 [8] on official controls for *Trichinella* in meat, which mandates wildlife surveillance in territories where wildlife–farm coexistence may affect the maintenance of *Trichinella*-free status.

The program is coordinated by the Emilia-Romagna Regional Authority and carried out through a structured network involving the Veterinary Services of the Local Health Units (Aziende USL), the Territorial Hunting and Fishing Departments (STACP), Provincial Police, Park Management Authorities, Territorial Hunting Areas (ATC), the Carabinieri Forestali, the Universities of Bologna and Parma, and Wildlife Rescue Centers (CRAS). This collaborative framework ensures systematic and standardised sample collection across multiple wildlife species and ecological contexts throughout the region, supporting consistent long-term monitoring of wildlife health threats.

### 2.2. Surveillance Targets and Sampling

During 2020–2024, the regional surveillance program includes targeted monitoring of *Trichinella* spp. to assess the potential risk of transmission from wildlife to domestic pigs. This approach supports food safety objectives by identifying and mitigating zoonotic threats at the interface between wild and domestic animal populations. The red fox was designated as the principal indicator species for *Trichinella* spp. surveillance due to its ecological role and wide distribution. Wild boars are routinely tested as part of mandatory food safety inspections, as well as when intended for personal consumption by hunters. Additional carnivorous species are included opportunistically in the surveillance program, provided that carcass condition is adequate for diagnostic sampling and analysis.

### 2.3. Diagnostic Procedures

A total of 104,338 wild mammals were examined for *Trichinella* spp. between 2020 and 2024 (Table 1). The species included wild boar (*Sus scrofa*), red fox (*Vulpes vulpes*), grey wolf (*Canis lupus*), European badger (*Meles meles*), stone marten (*Martes foina*), pine marten (*Martes martes*), European polecat (*Mustela putorius*), least weasel (*Mustela nivalis*), and golden jackal (*Canis aureus*).

Muscle samples were collected from carcasses submitted by hunters, wildlife recovery centers, or official wildlife control programs. For all animals, the preferred anatomical locations are the diaphragm, the hind-limb muscle, or the tongue. As alternative sites, muscles located near bones or tendons can be collected. According to our protocol, 5 g of tissue is sampled from the preferred anatomical sites, whereas 10 g is collected when alternative sites are used.

Muscle tissues were collected and examined for the presence of *Trichinella* spp. larvae using the magnetic stirrer-based artificial digestion method [9,10]. When larvae were detected, they were isolated, counted, and preserved in 90% ethanol. The preserved larvae were then submitted to the European Union Reference Laboratory for Parasites (EURLP) at the Istituto Superiore di Sanità (Rome, Italy) for molecular identification of *Trichinella* species. The preserved larvae were then submitted to the European Union Reference Laboratory for Parasites (EURLP) at the Istituto Superiore di Sanità (Rome, Italy) for species identification by multiplex PCR [11]. Briefly, DNA was purified from single larvae using a DNA IQ System kit (Promega, Madison, WI, USA) and a Tissue and Hair Extraction kit (Promega, WI, USA). Five primer sets, targeting specific regions (expansion segment V, ITS1, and ITS2) of the ribosomal DNA repeats, were used in multiplex PCR to obtain a species-specific electrophoretic DNA banding pattern [12,13].

## 3. Results

Among the animals tested, 12 individuals (0.011%) were found to be positive for *Trichinella* infection. Molecular analysis identified *T. britovi* in 11 cases, while one larval isolate could not be assigned to a specific species. Positive animals included seven wolves and five red foxes. No *Trichinella* larvae were detected in wild boar, which represented 96% of the samples. The number of animals tested per species and per year is summarised in Table 1. In Table 2, we report in detail the number of carcasses obtained from culled animals and those recovered as found dead. This distinction was possible only for wild boar and red fox, as all other species included in the study were exclusively represented by animals found dead. Annual prevalence values of *Trichinella* spp. for wolves and red foxes, including 95% Clopper–Pearson confidence intervals, are reported in Table 3.

**Figure 1:**
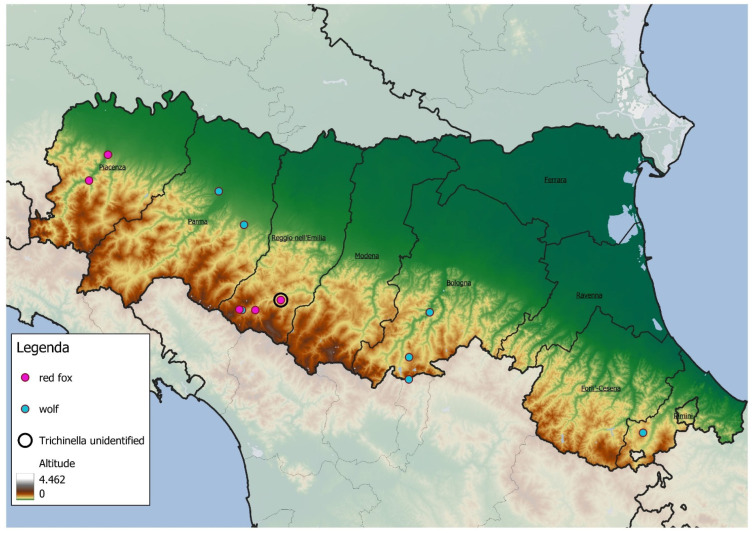
Map illustrating the geographic distribution of positive sample locations. Symbols indicate wolves and foxes that tested positive, while major geographic landmarks are included to facilitate spatial reference. Pink points represent red foxes, blue points represent wolves, and black circles indicate *Trichinella* samples for which the species was not identified. The legend also includes an altitude gradient to provide a sense of elevation across the study area.

The estimated age of positive animals is reported in Table 4. All positive samples were collected from individuals found in hilly areas or the lower Apennine mountain region, as shown in Figure 1.

## 4. Discussion

The results of this five-year surveillance program confirm the ongoing circulation of *Trichinella* spp. in wildlife populations within the Emilia-Romagna region, although at low prevalence. Among the 104,338 wild mammals tested, only 12 animals (0.011%) were positive, with *T. britovi* identified as the predominant species. Most positive animals (10/12) were adults and were found predominantly in hilly or Apennine areas. These findings are consistent with previous reports highlighting *T. britovi* as the most widespread *Trichinella* species among wild carnivores in continental Europe [1,14].

Recent estimates by the Large Carnivore Initiative for Europe suggest that the wolf population within the 27 EU Member States is approximately 19,000 individuals, with a broader European estimate nearing 21,500 [14]. In Italy, the wolf population is estimated at around 3300 individuals [15,16]. As apex predators, wolves are known to engage in scavenging behaviour [17], which may facilitate the persistence and spread of *Trichinella* spp. Their extensive territorial ranges, about 50–200 km^2^, and high mobility [18] could also support the introduction of these parasites into previously unaffected territories. According to the European Union One Health Zoonoses Report, 10.1% of wolves tested in Italy during 2022 were found to be infected with *Trichinella* spp. [19].

Large carnivores such as wolves, lynxes, and bears, due to their trophic position and longevity, are particularly susceptible to *Trichinella* spp. infections [17]. The low overall prevalence indicates that while *Trichinella* persists in the regional wildlife community, its circulation remains sporadic. This pattern may reflect ecological constraints, such as limited host density, trophic interactions, and spatial heterogeneity of suitable habitats. For example, large carnivores like wolves and lynxes are key hosts due to their scavenging behaviour and wide territorial ranges, but their low population densities likely limit their contribution to widespread transmission [20]. In contrast, red foxes, with high density and broad distribution, act as principal reservoirs, highlighting the differential epidemiological roles of sympatric species [3].

Recent ecological changes, including population growth of red foxes and wild boar, alongside the westward expansion of raccoon dogs (*Nyctereutes procyonoides*) and the spread of golden jackals (*Canis aureus*) into northwestern Europe, are likely to increase interspecies interactions and promote the transmission of *Trichinella* spp. [21,22]. These developments emphasise the need for dynamic and responsive wildlife surveillance systems that account for newly colonised regions and emerging host species.

The predominance of *T. britovi* among infected wolves and red foxes further supports their central role in the sylvatic transmission cycle of *Trichinella* spp. A critical biological trait contributing to the transmission of these nematodes is the extended viability of larvae within decomposing carcasses [1]. This adaptation increases the probability that scavengers will consume infected tissue, thereby perpetuating the parasite’s life cycle. The persistence of larvae post-mortem enhances the likelihood of successful transmission to subsequent hosts.

No larvae of *T. spiralis* were detected in the present study; only *T. britovi* was identified. This finding is consistent with the epidemiological scenario observed in Italy, where *T. spiralis* is only sporadically reported in wildlife. The parasite is primarily associated with domestic transmission cycles and remains the leading cause of human trichinellosis worldwide [5], yet its occurrence in wild animals is rare. In Italy, the first confirmed case in wildlife was documented in 1991 in a red fox from Bardonecchia, Piedmont, followed by isolated detections in 2016, 2017, and 2018 in red foxes from Travo, Emilia-Romagna [23,24]. More recently, *T. spiralis* was reported in a wolf from central Italy [25], raising potential concerns regarding its host range, as this species has not traditionally been considered a reservoir.

Although the wild boar is regarded as the principal sylvatic host for *T. spiralis*, such transmission events are usually confined to regions with limited veterinary control, often in association with political or economic instability [26,27]. Nonetheless, the proportion of *T. spiralis* among isolates from wild carnivores remains consistently low [4], in contrast to the more prevalent detection of *T. britovi*, as confirmed in this study.

**Figure 2:**
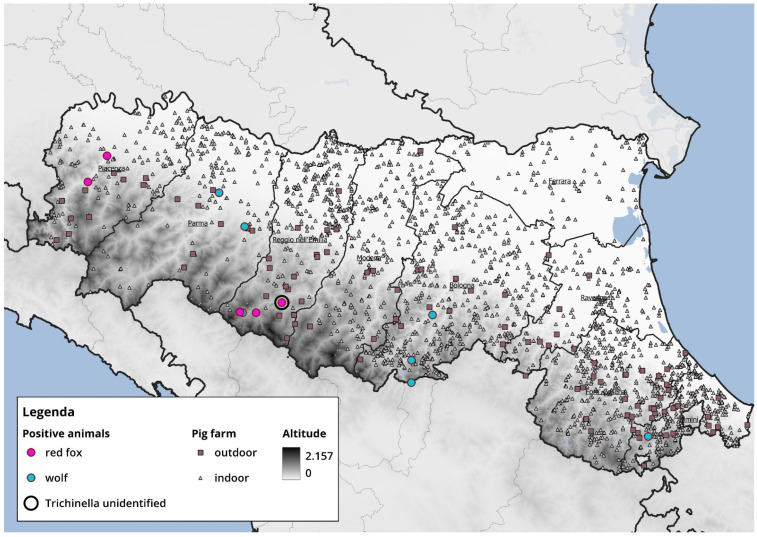
Map showing the spatial overlay between the locations of wild animals testing positive for *Trichinella* and the distribution of pig farms. Red dots represent infected red foxes, blue dots indicate infected wolves, grey squares denote semi-free-range pig, and grey triangles indicate intensive indoor pig farms.

Currently, there are no reported outbreaks in Italy of *Trichinella* in domestic pigs; however, wild boars represent a potential risk to pig farms, particularly those practicing organic or free-range farming. Wild boars serve as the primary sylvatic reservoir of *T. spiralis*, and their proximity to domestic pigs can facilitate parasite transmission, especially in farming systems with outdoor access and lower biosecurity. These circumstances emphasize the importance of stringent biosecurity measures to prevent parasite introduction at the wildlife–livestock interface. To this purpose, we have attempted to provide a map overlaying pig farms with the positive cases reported in this study (Figure 2). Positive samples were detected in areas located farther from pig farms; nevertheless, red foxes—and especially wolves—have wide home ranges, underscoring the importance of continuing targeted monitoring and surveillance in the coming years. It is important to highlight, however, that during the period covered by this study, African swine fever (ASF) was circulating in Italy; therefore, heightened attention and measures were implemented to strengthen farm biosecurity.

Our findings also highlight ecological and anthropogenic drivers of parasite distribution. In fact, in this study, infected animals were predominantly located in hilly and Apennine environments. This spatial pattern may reflect a combination of ecological and anthropogenic factors that promote parasite persistence, such as the availability of suitable hosts and human activities prevalent in these regions. While no clear association was observed between land-cover type and the presence of *T. britovi*, all our positive samples were detected in forested, semi-natural, and agricultural areas above 150 m. As noted by Pozio et al., elevation appeared to influence *Trichinella* species distribution [28]. This altitudinal trend has also been documented in previous studies from France, Italy, and Spain [2,28], and may reflect host-specific ecological preferences. *T. britovi* is more commonly associated with wild carnivores in upland habitats, whereas *T. spiralis* is more prevalent in domestic animals and wild boars inhabiting lowland regions. Furthermore, the enhanced cold tolerance of *T. britovi* may support its survival in higher elevation zones, with larvae capable of remaining viable in frozen carcasses for up to 11 months at −15 °C in carnivores and up to 3 weeks at −20 °C in swine, durations not tolerated by *T. spiralis*.

The absence of *Trichinella* spp. detection in wild boars in this dataset is particularly noteworthy, given their known function as both sentinel and bridge hosts connecting sylvatic and domestic cycles. This result may reflect a genuinely low prevalence in the local wild boar population, but alternative explanations include sampling limitations, dietary differences, or spatial heterogeneity in parasite distribution. Additionally, the feeding behaviour of wolves and red foxes in areas where small rodents, potential hosts of *Trichinella* spp., are abundant could support parasite persistence in these ecosystems.

The limited survival of *T. britovi* larvae in swine muscle may also explain the low prevalence detected in wild boars, with reported infection rates of approximately 0.02% in Italy and 2.5% in Latvia, despite much higher prevalence rates—up to 50% among wild carnivores [29,30].

Nonetheless, continued monitoring of wild boars remains critical from a food safety perspective, particularly given the zoonotic risk associated with consuming undercooked game meat. Although infrequent, cases of *T. pseudospiralis* infection in wild boar have been documented in past years [31]. Its low prevalence in the European Union (1.6%) suggests a limited epidemiological impact compared to other *Trichinella* species circulating within the region [32]. However, the regional wildlife health surveillance program recently identified *T. pseudospiralis* in a Western marsh harrier (*Circus aeruginosus*) [33], highlighting the need for further investigation into the role of avian species as potential reservoirs of this zoonotic parasite [34].

The detection of a *Trichinella* isolate that could not be identified to the species level likely reflects technical constraints, such as DNA degradation or a low larval count, which may compromise identification.

From a public health perspective, these findings emphasise the need for awareness campaigns directed at hunters, consumers of game meat, and individuals involved in wildlife handling. Promoting knowledge of the risks associated with consuming raw or undercooked game meat—especially from species known to harbour *Trichinella* larvae—is essential for reducing the likelihood of human infection. Additionally, proper carcass disposal practices must be encouraged to mitigate environmental contamination and interspecies transmission.

Overall, the low prevalence observed confirms that *Trichinella* spp. infections in wildlife in Emilia-Romagna are sporadic but persistently present. These data reinforce the value of long-term, structured surveillance programs in early detection and risk assessment, especially considering the potential for zoonotic transmission. Monitoring programs should remain responsive to changes in wildlife ecology and population dynamics, with an integrated approach that combines animal health, food safety, and public health perspectives.

## 5. Conclusions

The five-year surveillance program in Emilia-Romagna confirms the continued, low-prevalence circulation of *Trichinella* spp. in local wildlife, with *T. britovi* as the predominant species. Wolves and red foxes act as key sylvatic reservoirs, while wild boars currently play a limited role in regional transmission.

These findings emphasise the need for sustained multidisciplinary surveillance that integrates wildlife health, food safety, and public health. Surveillance systems should also remain adaptable to ecological and population changes to ensure timely detection and management of zoonotic risks posed by *Trichinella* spp.

## Figures and Tables

**Table 1:** Annual number of individuals recorded per species from 2020 to 2024. The table summarises the annual and cumulative counts of wild mammal species monitored within the study area over the five-year period.

Host Species	2020	2021	2022	2023	2024	Total
Wild-Boar (*Sus scrofa*)	19,866	26,596	19,457	18,355	16,155	100,429
Least Weasel (*Mustela nivalis*)	2	3	-	-	-	5
Stone Marten (*Martes foina*)	8	9	9	14	16	56
Grey Wolf (*Canis lupus*)	36	47	98	85	95	361
Pine Marten (*Martes martes*)	-	-	1	1	1	3
European Polecat (*Mustela putorius*)	-	1	1	1	-	3
Golden Jackal (*Canis aureus*)	-	1	7	1	9	18
European Badger (*Meles meles*)	22	63	96	103	130	414
Red fox (*Vulpes vulpes*)	389	550	662	644	804	3049
Total	20,323	27,270	20,331	19,204	17,210	104,338

**Table 2:** Number of carcasses collected per species during the study period, differentiated by acquisition source where available. For wild boar (*Sus scrofa*) and red fox (*Vulpes vulpes*), carcasses are separated into individuals culled during hunting activities and those recovered as found dead. For all other species (weasel, beech marten, wolf, pine marten, polecat, golden jackal, and badger), carcasses originate exclusively from animals found dead through passive surveillance. The dataset reflects heterogeneous sampling effort across space and time, largely influenced by hunting pressure, reporting frequency, and carcass detectability.

Species	Culled Animals	Animals Found Dead
Wild Boar (*Sus scrofa*)	98,798	1631
Weasel (*Mustela nivalis*)		5
Beech Marten (*Martes foina*)		56
Wolf (*Canis lupus*)		361
Pine Marten (*Martes martes*)		3
European Polecat (*Mustela putorius*)		3
Golden Jackal (*Canis aureus*)		18
European Badger (*Meles meles*)		414
Red Fox (*Vulpes vulpes*)	2908	141
Total	101,706	2632

**Table 3:** Prevalence (%) is calculated as (number of positive animals/number tested) × 100. Confidence intervals (95% CI) were computed using the Clopper–Pearson exact method. Wolf and red fox were the only species with sufficient annual sample sizes to allow reliable temporal prevalence estimates.

Year	Species	Tested	Positive	Prevalence (%)	95% CI
**2020**	Wolf (*Canis lupus*)	36	0	0.00	0–9.70
	Red fox (*Vulpes vulpes*)	389	1	0.26	0.01–1.40
**2021**	Wolf	47	0	0.00	0–7.50
	Red fox	550	1	0.18	0.00–1.00
**2022**	Wolf	98	1	1.02	0.03–5.60
	Red fox	662	2	0.30	0.04–1.10
**2023**	Wolf	85	3	3.53	0.70–10.00
	Red fox	664	1	0.15	0.00–0.80
**2024**	Wolf	95	2	2.11	0.26–7.40
	Red fox	804	0	0.00	0–0.46

**Table 4:** Identification of *Trichinella* species and larval burden in wild carnivores. The table reports, for each animal, the estimated age, the *Trichinella* species detected, and the larval density expressed as larvae per gram of muscle tissue, along with the geographic coordinates and altitude of the locations where positive animals were recovered (NA, data not available).

Host Species	Host Age Range	*Trichinella* Species Identification	Larvae Per Gram of Tissue	Geographic Coordinates (Lat. and Long.)	Altitude (m a.s.l.)
Wolf 1	Adult (>8 years)	*T. britovi*	5	44.65506 10.27828	226
Wolf 2	Adult (>2 years)	*T. britovi*	0.1	4.7763 10.15384	117
Wolf 3	Adult (>2 years)	*T. britovi*	NA	44.32786 11.19801	155
Wolf 4	Adult (>2 years)	*T. britovi*	NA	44.09012 11.08524	1175
Wolf 5	Adult (>2 years)	*T. britovi*	NA	44.16919 11.08918	690
Wolf 6	Subadult (1–2 years)	*T. britovi*	76	43.87243 12.23744	777
Wolf 7	Subadult (1–2 years)	*T. britovi*	0.3	44.35007 10.26132	1045
Red Fox 1	Young (<1 year)	*T. britovi*	1	44.81946 9.50189	206
Red Fox 2	Adult (>3 years)	*T. britovi*	12.2	44.91149 9.59787	145
Red Fox 3	Adult (>2 years)	Not detectable	0.3	44.3836 10.45598	631
Red Fox 4	Adult (>2 years)	*T. britovi*	0.3	44.35212 10.24844	1120
Red Fox 5	Adult (>2 years)	*T. britovi*	0.3	44.34929 10.32803	711

## Data Availability

Data are contained within the article.
